# A Droplet-Based Microfluidic Platform for High-Throughput Culturing of Yeast Cells in Various Conditions

**DOI:** 10.3390/mi15081034

**Published:** 2024-08-15

**Authors:** Min-Chieh Yu, Yung-Shin Sun

**Affiliations:** Department of Physics, Fu-Jen Catholic University, New Taipei City 24205, Taiwan; 409290549@m365.fju.edu.tw

**Keywords:** microfluidics, micro-droplet, high-throughput, yeast, cycloheximide

## Abstract

Yeast plays a significant role in a variety of fields. In particular, it is extensively used as a model organism in genetics and cellular biology studies, and is employed in the production of vaccines, pharmaceuticals, and biofuels. Traditional “bulk”-based studies on yeast growth often overlook cellular variability, emphasizing the need for single-cell analysis. Micro-droplets, tiny liquid droplets with high surface-area-to-volume ratios, offer a promising platform for investigating single or a small number of cells, allowing precise control and monitoring of individual cell behaviors. Microfluidic devices, which facilitate the generation of micro-droplets, are advantageous due to their reduced volume requirements and ability to mimic in vivo micro-environments. This study introduces a custom-designed microfluidic device to encapsulate yeasts in micro-droplets under various conditions in a parallel manner. The results reveal that optimal glucose concentrations promoted yeast growth while cycloheximide and Cu^2+^ ions inhibited it. This platform enhances yeast cultivation strategies and holds potential for high-throughput single-cell investigations in more complex organisms.

## 1. Introduction

Yeast is a microorganism that holds great importance in various aspects of human life, particularly in the fields of food production, biotechnology, and scientific research. For example, yeast is widely used in the baking industry to make bread, pastries, and other baked goods. It is also essential for the fermentation of sugars to produce alcohol in the brewing and winemaking industries. In the field of biotechnology, yeast is frequently used as a model organism for research, and it has contributed significantly to our understanding of genetics and cellular biology [1,2,3]. Yeast cells are eukaryotic, like human cells, making them a useful model for studying complex cellular processes. It can also be used in the production of various vaccines and pharmaceuticals. The hepatitis B vaccine is produced using yeast cells that have been genetically modified to produce viral proteins [4]. Moreover, yeast plays a vital role in the production of biofuels, such as bioethanol, by converting sugars and starches from plants into ethanol through fermentation, offering a more sustainable and environmentally friendly alternative to fossil fuels [5,6]. For environmental and industrial applications, yeast can help in the treatment of certain types of wastewaters by aiding in the breakdown of organic matter, as well as in the production of enzymes, vitamins, organic acids, biodegradable plastics, and other sustainable materials [7,8]. Several factors have been identified to affect the growth of yeast, including nutrient availability, pH level, temperature, salinity, light intensity, and ionic and CO_2_ concentrations [9,10,11]. Therefore, to optimize the usage of yeast and maximize yeast-derived products, it is crucial to fine-tune their growth conditions. Traditionally, yeast studies have employed “bulk”-based methods that provide average effects, essentially aggregating responses across a large cell population. For example, adaptive laboratory evolution (ALE) experiments demand significant laboratory resources and consume considerable time [12,13]. They necessitate the assessment of growth kinetics within intermediate populations and the screening of numerous candidate strains to identify an optimal strain exhibiting enhanced phenotypic traits. This evolutionary process may span hundreds of generations, and the conventional growth evaluation, relying on optical density (OD) measurements, must be executed with three repetitions for each population or strain. However, these “bulk”-based experiments overlook the inherent variability among cells. Studies have shown that even genetically identical cells under identical culture conditions can exhibit substantial differences in both molecular composition and physical traits [14]. Consequently, it is essential to culture and monitor individual cells or small cell groups to explore how the stochastic nature of individual cells influences the behavior of the entire population.

Micro-droplets refer to tiny liquid droplets that are typically in the micrometer range in size. Due to their unique properties and capabilities, these miniature droplets find various applications in scientific, industrial, and technological fields. They can be used to encapsulate and deliver medications with precision, allowing for controlled release and targeted therapy [15]. Also, micro-droplets are instrumental in various biological and biochemical applications, such as PCR (polymerase chain reaction) for DNA amplification and cell manipulation [16]. Most importantly, these minimized compartments are frequently employed in high-throughput experimentation, enabling researchers to simultaneously test numerous experimental conditions, accelerating the discovery of new compounds and the sorting of cells, microorganisms, and particles [17,18]. Due their small sizes, micro-droplets serve as efficient carriers for analyzing small quantities of substances, facilitating precise measurements and reducing reagent consumption. Furthermore, they offer a variety of advantages, including uniform size, independence, compact dimensions, and high surface-area-to-volume ratio. All these characteristics make micro-droplets an ideal platform for conducting single-cell-based studies [19,20]. When one or few cells are cultured within these droplets, they effectively mimic three-dimensional (3D) in vitro micro-environments [21]. For example, in single-cell analysis, the dispersed fluids containing individual cells can serve various purposes, such as acting as a culture medium containing antibodies for the screening of cells secreting specific biomolecules, a lysis buffer for DNA sequencing, or a culture medium infused with drugs for drug library screening [22,23]. The isolation of single cells within these micro-droplets allows them to release detectable biomolecules, greatly expediting their identification through techniques like fluorescence-activated cell sorting (FACS) [24]. Micro-droplet-based systems have been employed to investigate a wide array of microorganisms, including algae, bacteria, and even *Caenorhabditis elegans* (*C. elegans*) [25,26].

Microfluidic devices, incorporating micro-fabricated structures and fluidic components, offer several distinct advantages including reduced volume, cost-effectiveness, and ease of fabrication. This is particularly valuable in cell-related research where minimal amounts of cells and reagents are required compared to traditional cell culture dishes and microplates [27]. Notably, these in vitro devices serve as an ideal platform for mimicking the fluid-circulating micro-environment encountered by in vivo tissue cells, exposed to various fluidic stresses such as blood and bodily fluids. Furthermore, these compact microfluidic devices enable precise and controllable application of diverse chemical and physical stimuli to cells, facilitating investigations into their responses. For instance, collective cell migration, including phenomena like chemotaxis, electrotaxis, and phototaxis, has been extensively explored within microfluidic setups [28,29,30]. In the context of micro-droplet generation, micro-scaled aqueous droplets are introduced into an oil stream within a micro-sized channel. Within microfluidic devices, droplets can be generated through either passive or active methods [31]. Passively, techniques like flow-focusing, co-flow, or cross-flow generators are employed to introduce the dispersed fluid (e.g., water) into the continuous fluid (e.g., oil) using mechanisms such as squeezing, dripping, jetting, tip-streaming, and tip-multi-breaking modes [32,33]. In these cases, both the dimensions of the channels and the flow rates of the aqueous and oil phases play crucial roles in determining droplet size and generation rates [34,35].

However, in nearly all studies, micro-droplets generated within microfluidic devices exhibit monodispersity, implying uniform conditions within each droplet. This characteristic severely constrains the capacity to efficiently screen different culture media and explore their impact on cell growth. To address this limitation, this study introduces a custom-designed microfluidic device capable of generating micro-droplets for encapsulating yeasts under varying concentrations of glucose, cycloheximide, and Cu^2+^. Over a monitoring period of 8 h, we observed the growth of yeasts *Saccharomyces cerevisiae* (*S. cerevisiae*) within these distinct micro-environments. The results demonstrate that, in general, the presence of optimal glucose concentrations fostered the growth of yeasts, whereas the presence of cycloheximide and Cu^2+^ in the medium inhibited their growth. These insights provide valuable guidance for enhancing the cultivation of yeast for use in food industry, biotechnology, biofuel production, and a variety of scientific research. Moreover, this platform can be extended to facilitate high-throughput investigations of more complex animal and human cells at the single-cell level.

## 2. Materials and Methods

### 2.1. Chip Design and Fabrication

**Figure 1:**
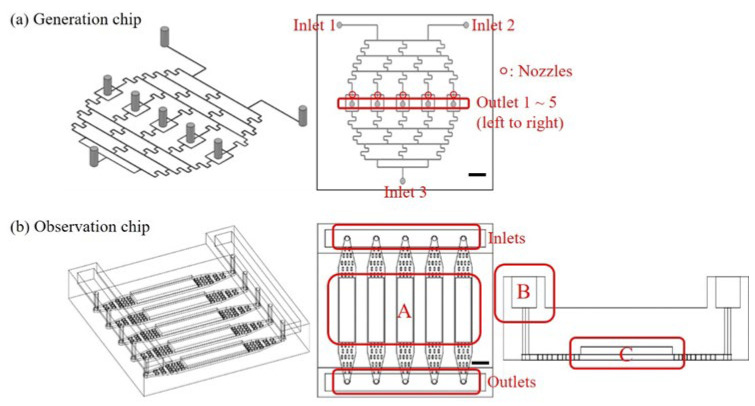
Designs of the (**a**) generation and (**b**) observation chips. Scale bar = 1 cm. In (**b**), A: observation area; B: raised reservoir for oil storage; C: elevated space for droplet storage.

Two separate chips were designed and fabricated: the generation chip for generating yeast-encapsulated droplets and the observation chip for observing the growth of yeasts in real time. AutoCAD (Autodesk, San Francisco, CA, USA) was used to create the designs for mask production, while the devices were manufactured using the standard soft lithography technique. The mask, having a dimension of 10 cm × 10 cm, was printed with a resolution of 20,000 dpi. First, a negative mode of SU-8 GM1070 (EM RESIST LTD., Macclesfield, UK) was applied to a 4 inch silicon wafer, and the wafer was spun at 2000 rpm for 45 sec. The mode was formed after sequential soft-bake, UV-exposure, post-bake, development, fixing, and hard-bake. Then, PDMS (Dow Corning, Midland, MI, USA) mixed with a curing agent in a 10:1 ratio was used to replicate the mold, forming the microstructures. This process was carried out under vacuum with a 75 °C, 90 min bake. The PDMS layer was then bonded onto a glass slide via O_2_-plasma activation at 18 W for 40 s under an O_2_ pressure of 600 mTorr (Harrick Plasma, Ithaca, NY, USA) to create the integrated chip. Figure 1a shows the design of the generation chip. The microfluidic channels’ height and width (excluding the inlets, the outlets, and the nozzles where two phases meet) were set at 55 and 200 μm, respectively. The size of the nozzles was 60 μm. The dispersed phase, resembling the “Christmas tree” geometry [36,37], initiated with two inlets: one with the maximum concentration of 1 (inlet 1 in Figure 1a) and the other with the minimum concentration of 0 (inlet 2 in Figure 1a). Following the first separation and mixture, concentrations of 1, 0.5, and 0 could be achieved. Subsequently, the third separation and mixture resulted in concentrations of 1, 0.875, 0.5, 0.125, and 0 before entering the nozzles. Here, the assumption is made that all liquids split-flow smoothly and equally around the fork. After flowing in the continuous phase from inlet 3 (see Figure 1a), this design enabled the generation of micro-droplets containing five different concentrations of the medium. The generation chip’s outlets (outlet 1~5 in Figure 1a) were tubed to the observation chip’s inlets (see Figure 1b) for collecting the droplets for long-term, real-time observation. As depicted in Figure 1b, the observation chip comprised five identical channels, with the designated observation area highlighted by a red rectangle A. The side view of one of these channels is illustrated in the rightmost panel of Figure 1b. Each channel featured a raised reservoir at its entrance and exit (part B in Figure 1b) to store oil (Novec 7500 + dSURF, as detailed in the subsequent section). This served to prevent droplet shrinkage resulting from oil evaporation. Notably, the droplets were confined within the elevated space (part C in Figure 1b) due to their lower density compared to that of the oil.

### 2.2. Yeast Preparation and Chemicals

Having simple and well-understood genetics, *S. cerevisiae* yeasts are extensively used as model eukaryotic organisms in biological research. They are typically spherical or oval in shape, measuring a few μm in diameter. Before experiments, yeasts were cultured in YPD (Yeast Extract Peptone Dextrose) broth containing 1% *w*/*v* yeasts extract, 2% *w*/*v* bacteriological peptone, and 2% *w*/*v* dextrose (glucose). YP (YPD without dextrose) broth was also used to study the effects of glucose on the growth of yeasts. Cycloheximide was dissolved in YPD broth to a desired concentration of 1 mg/mL. This chemical is a natural product and a well-known antibiotic and fungal toxin. It is derived from the bacterium *Streptomyces griseus*, and is used in various fields, including biology and medicine, for its ability to inhibit protein synthesis in both prokaryotic and eukaryotic cells. It does this by interfering with the translocation step in the process of ribosomal translation [38,39]. By binding to the ribosome, it prevents the elongation of a growing polypeptide chain during protein synthesis. CuSO_4_·5H_2_O was dissolved in YPD broth to a desired Cu^2+^ concentration of 200 μM.

### 2.3. Experimental System and Procedure

**Figure 2:**
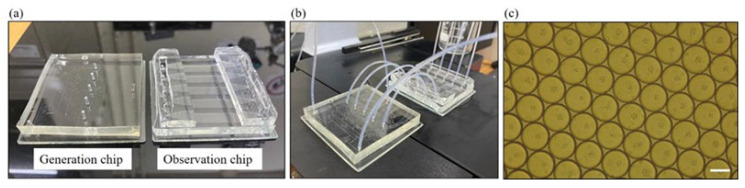
Pictures showing (**a**) the generation and observation chips, (**b**) the two chips connected, and (**c**) the yeast-encapsulated droplets (scale bar = 100 μm).

Figure 2a shows a picture of the generation and observation chips. Before experiments, these devices were washed with 1× phosphate buffered saline (PBS) then oil to make sure they were clean enough and not blocked by any unwanted residues. As depicted in Figure 1a, the flow-focusing geometry serves as the mechanism for generating micro-droplets. Initially, the nozzle was filled with the continuous-phase liquid. Subsequently, the dispersed-phase liquid approached the junction, establishing the immiscible interface. Through the continuous flow of both liquids, the interface was disrupted, leading to the formation of uniform droplets. The continuous-phase inlet (inlet 3 in Figure 1a) was connected to a syringe containing a mixture (Fluigent, Le Kremlin-Bicêtre, France) with a weight-to-weight ratio of 98% 3M™ Novec™ 7500 fluorinated oil and 2% dSURF, a high-performance surfactant utilized to create the water–oil interface in the micro-droplets. The two dispersed-phase inlets (inlet 1 and 2 in Figure 1a) were connected to syringes containing suspensions of yeasts in desired culturing media. The initial concentrations of the yeast suspensions were checked by a spectrophotometer (U-5100, Hitachi, Tokyo, Japan) to make sure that they had almost the same OD (optical density) values of around 1.4. This value corresponds to roughly 2.1 × 10^7^ cells/mL of yeasts. To explore their impacts on yeast growth, various concentrations of glucose, cycloheximide, and Cu^2+^ (in CuSO_4_·5H_2_O) were prepared. For instance, by using two dispersed liquids with Cu^2+^ concentrations of 0 and 200 μM, concentrations ranging from 0, 25, 100, 175 to 200 μM were achieved within micro-droplets across five outlets. The initial concentration of yeasts was also varied to investigate its effects on their growth. Syringe pumps (New Era, Farmingdale, NY, USA) were employed to control flow rates, with 400 and 100 μL/min set for the continuous and dispersed inlets, respectively. After flowing in all liquids for a few minutes, stable micro-droplets were generated and then transferred into five separate culturing channels of the observation chip through Teflon tubes for long-term storage, as shown in Figure 2b. Figure 2c exhibits a picture of the yeast-encapsulated droplets. The observation chip was mounted on top of an inverted microscope (ESPA SYSTEMS Co., Hsinchu, Taiwan) for capturing bright-field images under a 100× magnification.

### 2.4. Data Analysis

The growth of yeasts under diverse culture conditions was monitored by capturing images of the five channels of the observation chip at a 1 h interval over a period of 8 h. At each time point for every channel, a minimum of two pictures were taken, each containing a maximum of 40 micro-droplets. For each testing medium, two independent experiments were performed. To carry out statistical analysis, for a specific channel (under a specific culture condition) at one specific time point, at least 10 droplets having similar initial yeast numbers were selected from two independent experiments. Since yeasts tended to stick together, it was difficult to count individual yeasts. Instead, the yeast-covered area in each droplet was quantified using ImageJ software (ver. 1.54 g) from the National Institute of Health, USA. Mean values, along with standard deviations (SDs), were subsequently computed over at least 10 droplets for a specific medium condition at a given time. These values were plotted in a time-dependent manner after normalized to the initial (time = 0 h) ones. Two-tailed, unpaired (homoscedastic) *t*-tests were performed, with *p*-values less than 0.05 considered statistically significant.

## 3. Results and Discussion

### 3.1. Droplet Characterization

**Figure 3:**
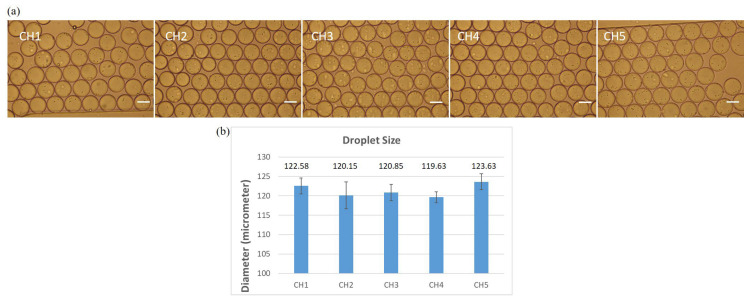
(**a**) Images of droplets in five channels of the observation chip. (**b**) Mean diameters with SDs of the droplets in these channels.

Images of droplets within five individual channels of the observation chip are shown in Figure 3a. The diameters of these droplets (at least 10 droplets were selected in each channel) were analyzed using ImageJ and indicated in Figure 3b. In general, uniform droplets were generated across all channels, with an overall diameter of approximately 120.14 ± 2.47 μm. Individually, the mean diameters with SDs of the droplets in five channels were about 122.58 ± 2.06, 120.15 ± 3.46, 120.85 ± 2.09, 119.69 ± 1.41, and 123.63 ± 2.07 μm.

### 3.2. Yeast Growth

Yeast undergoes distinct growth phases when cultured in a suitable medium. These phases reflect the changes in population size and metabolic activity over time. The main growth phases of yeast include lag, exponential, stationary, and death phases [40,41]. In the lag phase (typically 1~6 h), cells are metabolically active but not dividing rapidly. They are synthesizing essential enzymes, proteins, and other molecules required for growth. In the exponential phase (typically 6~24 h), cell division occurs at a constant and maximum rate, leading to a rapid increase in cell number. The metabolic activity is high, and cells are most uniform in terms of physiological properties. In the stationary phase, the number of new cells produced is roughly equal to the number of cells dying. Metabolic activities shift to maintenance and survival, with some cells entering a quiescent state. In the final phase, the population decreases as cells deplete remaining nutrients and are increasingly affected by toxic waste products. In the present study, with enough nutrients supplied, yeasts are expected to grow in the exponential phase. In this phase, an exponential equation $N_{2}/N_{1}=e^{(t_{2}-t_{1})\mu }$ is used to fit the growth curve to obtain the growth rate *μ* for a specific culture condition [40]. In this equation, *N*_2_ and *N*_1_ are the numbers of yeasts at time *t*_2_ and *t*_1_, respectively.

### 3.3. Effects of Glucose on the Growth of Yeasts

**Figure 4:**
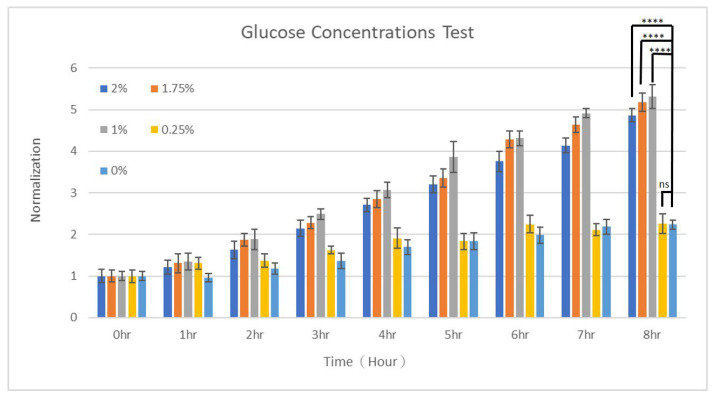
The growth of *S. cerevisiae* cells when cultured in different concentrations of glucose. *T*-tests were performed with ns: not significant difference (*p* > 0.05), * *p* < 0.05, ** *p* < 0.01, *** *p* < 0.001, and **** *p* < 0.0001.

Glucose is indispensable for the optimal growth, metabolism, and survival of yeasts. It is the primary energy source for yeasts: through glycolysis, it is broken down to produce adenosine triphosphate (ATP), providing energy currency to various cellular processes including growth and reproduction [42]. Moreover, glucose serves as a key carbon source for yeasts to build cellular components such as proteins, lipids, and nucleic acids [43]. A variety of yeasts, especially *S. cerevisiae*, can ferment glucose anaerobically to produce ethanol and carbon dioxide, which is the basis for industries such as brewing, baking, and bioethanol production [44]. Glucose can also regulate various metabolic pathways in yeasts, including the cell cycle, and affect their ability to respond to environmental stress [45,46]. In this study, the effects of glucose concentration on the growth of yeasts were investigated by using two dispersed YP media containing glucose concentrations of 0 and 2%, resulting in five distinct concentrations of 0, 0.25, 1, 1.75, and 2% in the generated micro-droplets across five outlets. The growth of yeasts was monitored for 8 h in 1 h intervals. After analyzing the yeast-covered area over time for each condition, the data were normalized to the starting points. Figure 4 shows these yeasts grew in a time-dependent manner. As illustrated, *S. cerevisiae* cells grew very slowly without or with just a little supply of glucose. Statistical significance was found between groups with glucose higher than 1% and without glucose. The numbers of yeasts merely doubled when they were not supplied with glucose or supplied with only 0.25% of it. As the concentration increased to and beyond 1%, the numbers after 8 h of culture were five times larger compared to initial values. By fitting these data to the exponential equation, the growth rates were about 0.092, 0.1026, 0.203, 0.1947, and 0.1815 h^−1^ (h^−1^) for glucose concentrations of 0, 0.25, 1, 1.75, and 2%, respectively. The values of R^2^ for these fittings were 0.899, 0.898, 0.957, 0.968, and 0.965, respectively, indicating that the exponential model fitted well to the growth of yeasts. These yeasts, under the present culture conditions, were growing in the exponential phase. Unexpectedly, a concentration of 1%, instead of 2%, resulted in the optimal (largest) growth rate. The influence of glucose on yeast growth was usually studied in a wide range of concentrations. For example, Ramos-Gomez et al. investigated the effect of glucose concentration on *S. cerevisiae* growth under three levels of glucose: 0.5% glucose represented a low level (caloric restriction), 2% corresponded to a standard level, and 10% was a high level [47]. It was found that the growth was only slightly faster in 2% glucose (growth rate μ ~ 0.2379 h^−1^) compared to that in 10% glucose (μ ~ 0.2019 h^−1^), while at 0.5% glucose, *S. cerevisiae* cells exhibited the slowest growth (μ ~ 0.1602 h^−1^) [47]. These values are close to what were obtained in this study, both suggesting that, in general, growth rates of approximately 0.2~0.23 h^−1^ could be attained with 1~2% of glucose supply. In another study, Ziv et al. modeled the growth rate of *S. cerevisiae* cells in response to glucose concentration as a continuous function [48]. The glucose concentration was varied from 0.05 to 4 mM, and the growth rate changed correspondingly from 0.1 to 0.4 h^−1^, with a saturation occurred beyond 2 mM [48]. Different strains of yeasts were shown to exhibit slower growth and lower maximum densities when cultured in high (10%) to extremely high (60%) glucose concentrations [49]. For example, in the *S. apicola* strain, the maximum densities after 80 h of culture were the highest (2.7 × 10^7^ cells/µL) and the lowest (9 × 10^6^ cells/µL) in 2 and 60% glucose, respectively, and the duration of the exponential phase decreased with increasing glucose concentration, being 18 and 12 h at concentrations of 2 and 60%, respectively [49].

### 3.4. Effects of Cycloheximide on the Growth of Yeasts

**Figure 5:**
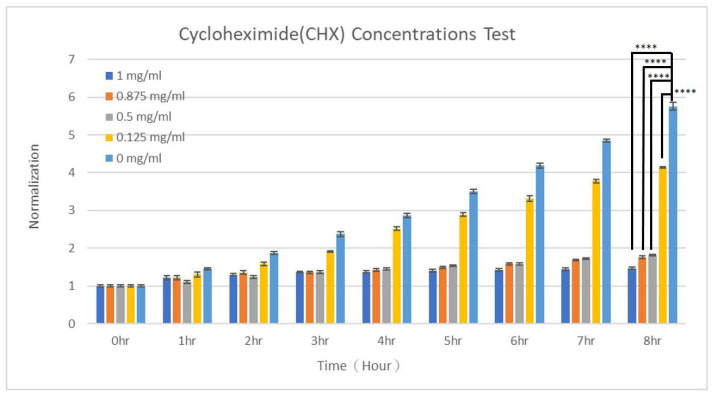
The growth of *S. cerevisiae* cells when cultured in different concentrations of cycloheximide. *T*-tests were performed with ns: not significant difference (*p* > 0.05), * *p* < 0.05, ** *p* < 0.01, *** *p* < 0.001, and **** *p* < 0.0001.

Cycloheximide is a potent inhibitor of yeast growth due to its effect on protein synthesis, leading to growth arrest, cell cycle disruption, and potentially apoptosis [50]. Due to the inhibition of protein synthesis, yeast cells cannot produce the proteins necessary for various cellular processes, leading to their ceased dividing and expanding. Moreover, yeasts often rely on the synthesis of specific stress response proteins to survive in adverse conditions, and cycloheximide treatment can impair the ability of yeast cells to respond to stress, making them more susceptible to environmental challenges [51]. In the present work, to evaluate how cycloheximide affects the growth of *S. cerevisiae* cells, 0 and 1 mg/mL of cycloheximide in YPD media were flowed into the two inlets of the dispersed-phase channels to attain concentrations of 0, 0.125, 0.5, 0.875, and 1 mg/mL in five sets of yeast-encapsulated droplets. The normalized growth curves are indicated in Figure 5. *T*-tests showed statistical significance between groups with and without the addition of cycloheximide. As seen, under concentrations higher than 0.5 mg/mL, the yeast grew quite slowly with numbers after 8 h being only 1.82, 1.76, and 1.46 times larger compared to initial values under concentrations of 0.5, 0.875, and 1 mg/mL, respectively. When the concentration was reduced to 0.125 mg/mL, a 4.14-fold growth was observed, still less than the control (without cycloheximide) of 5.76 times. These curves were then fitted to the exponential curve to obtain the growth rates, giving values of 0.0522, 0.0673, 0.0682, 0.1659, and 0.201 for concentrations of 1, 0.875, 0.5, 0.125, and 0 mg/mL, respectively. The R^2^ values of for these fittings were 0.569, 0.906, 0.97, 0.973, and 0.98, respectively, suggesting that these yeasts, except those exposed to 1 mg/mL of cycloheximide, were growing in the exponential phase. These findings suggest that low concentrations of cycloheximide (less than 0.125 mg/mL) did not much affect the growth of *S. cerevisiae* cells, but concentrations above a critical value (0.5 mg/mL) could significantly inhibit their growth. Doyle et al. also found that the growth of *P. pastoris* yeasts was hindered in the presence of cycloheximide, in a dose-dependent manner from 0 to 0.2 mg/mL [52]. The concentration of cycloheximide necessary to inhibit 50% of the growth of *S. pastorianus* yeasts (ED_50_) was determined to be as low as 0.018 mg/mL when the inoculum was 0.022 mg/mL [53]. Cycloheximide was commonly used as a protein synthesis inhibitor to study how yeast cells’ response to stress was affected. For example, Ribeiro et al. investigated the impact of cycloheximide on the survival rates of *S. cerevisiae* cells treated with metal (iron, copper, and manganese) complexes [54]. It was found that in the presence of 0.5 mg/mL cycloheximide, the metal compounds continued to be highly effective, as evidenced by the high survival rates of *S. cerevisiae* cells whose protein synthesis is blocked by cycloheximide [54]. The influence of cycloheximide on the toxicity on *S. cerevisiae* cells was investigated, showing that its presence reduced the loss of cell viability induced by 750 and 1000 μmol/L of lead (Pb) [55]. This implied that the loss of viability induced by Pb was dependent on protein synthesis [55].

### 3.5. Effects of Cu^2+^ on the Growth of Yeasts

**Figure 6:**
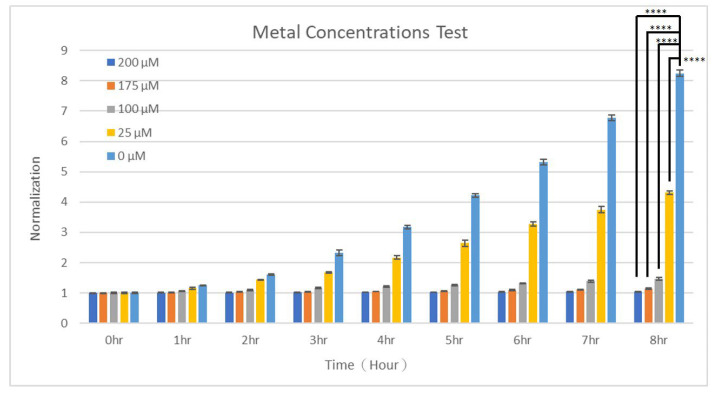
The growth of S. cerevisiae cells when cultured in different concentrations of Cu^2+^. *T*-tests were performed with ns: not significant difference (*p* > 0.05), * *p* < 0.05, ** *p* < 0.01, *** *p* < 0.001, and **** *p* < 0.0001.

Heavy metals can have various effects on the growth of yeasts, often depending on the type and concentration of the metal involved. For example, Cu, Pb, mercury (Hg), and arsenic (As) are toxic to yeasts even at low concentrations [56,57]. They may also induce oxidative stress in yeast cells by generating reactive oxygen species (ROS), inhibit the activity of crucial enzymes by binding to their active sites, cause mutations by inducing DNA breaks or cross-linking, and disrupt cellular membranes [58]. Some strains, such as *S. cerevisiae*, can adapt to certain heavy metals through gene expression changes, including the upregulation of metal transporters and antioxidant proteins [59]. By using the present microfluidic device, the effects of Cu^2+^ from 0 to 200 μM on the growth of *S. cerevisiae* cells were studied. Figure 6 shows the growth curves of *S. cerevisiae* cells under different concentrations of Cu^2+^. Statistical significance was observed between groups in the presence and absence of Cu^2+^. In the presence of Cu^2+^ from 100 to 200 μM, the growth was strongly inhibited. After 8 h of culture, the numbers of yeasts remained almost the same at [Cu^2+^] = 175 and 200 μM and increased to only around 1.5 times comparing to the start point at [Cu^2+^] = 100 μM. When the concentration was reduced to 25 μM, a 4.3-fold increase in the number was observed, suggesting still noticeable growth inhibition compared to an 8.2-fold increase without any Cu^2+^. After fitting these curves, growth rates of 0.005, 0.0124, 0.0402, 0.1585, and 0.2304 h^−1^ were obtained for Cu^2+^ of 200, 175, 100, 25, and 0 μM, respectively. This exponential model fitted well to all growth curves, provided that the R^2^ values were 0.977, 0.93, 0.971, 0.958, and 0.962, respectively. These results indicate Cu^2+^ higher than 100 μM could severely inhibit the growth of *S. cerevisiae* cells, and even a lower concentration of 25 μM could still suppress their growth to some extent. Soares et al. studied the toxic effects induced by heavy metals in the yeast *S. cerevisiae*, finding the decreasing order of toxicity to be copper, lead, and nickel (Ni) [56]. The presence of 200 μM of heavy metal caused a decrease in the number of viable cells by about 50% in the first 5 min for Cu and in 4 h for Pb, whereas Ni up to 200 μM exhibited no toxic effects over a culture period of 48 h [56]. It was also found that prior to Cu treatment, the addition of 0.5 mM calcium reduced the loss of cell viability via decreasing the release of UV-absorbing compounds [56]. Wang et al. examined the effects of Cu^2+^ at high concentrations of 0.5, 1.0, and 1.5 mM on the growth, surface characteristics, and elasticity of two strains of *S. cerevisiae* [60]. The findings indicated that elevated levels of Cu^2^⁺ slowed the growth rate of *S. cerevisiae* cells and decreased the utilization of reducing sugars [60]. Moreover, after Cu^2^⁺ treatment, the cells exhibited intercellular adhesion, became smaller and deformed, and their surfaces appeared uneven and rough [60]. Adamo et al. investigated the molecular and physiological factors that enable yeast strains to tolerate copper [61]. The results showed that while the growth of *S. cerevisiae* cells was inhibited at [Cu^2+^] = 3.125 mM, *Candida humilis* yeasts tolerated up to 6.25 mM of Cu^2+^ due to high constitutive levels of superoxide dismutase and catalase [61]. And, after undergoing evolution, both strains were able to proliferate in up to 15.625 mM of Cu^2+^ and accumulate high levels of intracellular Cu [61].

### 3.6. Effects of Initial Concentration on the Growth of Yeasts

**Figure 7:**
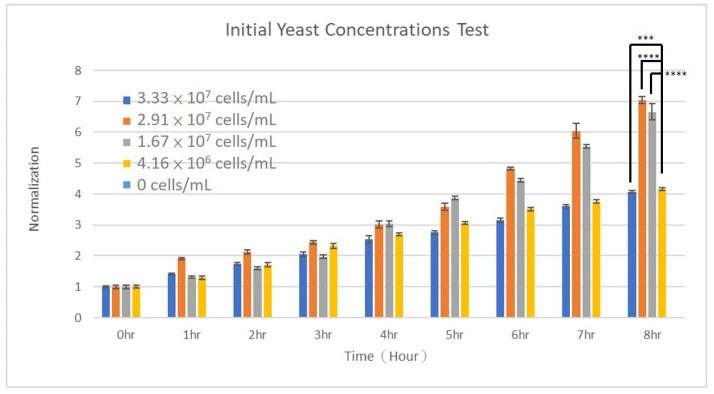
The growth of S. cerevisiae cells when cultured in different initial concentrations. *T*-tests were performed with ns: not significant difference (*p* > 0.05), * *p* < 0.05, ** *p* < 0.01, *** *p* < 0.001, and **** *p* < 0.0001.

The initial concentration of yeast cells can significantly impact their growth dynamics, which were seldom investigated. At low initial concentrations, yeasts often experience an extended lag phase during which they acclimate to the new environment, synthesizing necessary enzymes and adjusting their metabolism. Therefore, the overall population density may increase more slowly compared to the optimal initial concentration. On the other hand, high initial concentrations lead to intense competition for nutrients, potentially resulting in nutrient depletion and accumulation of metabolic byproducts that can inhibit growth. In this case, the yeast population may reach the stationary phase sooner. At an optimal initial concentration, yeasts can quickly transition from the lag phase to the exponential growth phase, ensuring that there is a sufficient number of cells to rapidly utilize nutrients and produce growth factors that benefit the entire population. In addition to the strain of yeast, the optimal initial concentration depends on a variety of factors including nutrient concentration, oxygen availability, pH levels, stress responses, and so on. With the present high-throughput microfluidic chip, the initial yeast concentration was varied to study how it affects the growth dynamics. Yeast suspension in YPD medium, with a measured OD value of 2.22, and YPD broth only were flowed into the two inlets of the dispersed-phase channels. This OD value corresponds to around 3.33 × 10^7^ cells/mL of yeasts. This led to five different initial concentrations: 0, 4.16 × 10^6^, 1.67 × 10^7^, 2.91 × 10^7^, and 3.33 × 10^7^ cells/mL. The growth curves of *S. cerevisiae* cells under these initial concentrations are shown in Figure 7. Clearly, the growth of *S. cerevisiae* cells depended on its initial density. At a low concentration of 4.16 × 10^6^ cells/mL, the number of yeast cells increased approximately 4.15 times over 8 h of culture. Over the same period, a 4.06-fold growth was observed at a high concentration of 3.33 × 10^7^ cells/mL. At intermediate concentrations of 1.67 × 10^7^ and 2.91 × 10^7^ cells/mL, 6.66- and 7.04-fold increases in the numbers were observed, respectively. Statistical significance was found between groups with optimal (1.67 × 10^7^ and 2.91 × 10^7^ cells/mL) and low (4.16 × 10^6^ cells/mL) or high (3.33 × 10^7^ cells/mL) initial concentrations. The growth rates were fitted to be 0.1731, 0.2089, 0.2219, and 0.1644 h^−1^ as the initial concentration increased from 4.16 × 10^6^ to 3.33 × 10^7^ cells/mL, with computed R^2^ values being 0.946, 0.963, 0.969, and 0.968. Again, these good fitting indicated yeasts under the present conditions were growing in the exponential phase. Based on these results, initial concentrations of 4.16 × 10^6^ and 3.33 × 10^7^ cells/mL were considered slightly low and high, respectively, in comparison with the optical initial concentration in the range of 1.67 × 10^7^ to 2.91 × 10^7^ cells/mL. An initial concentration of 2.1 × 10^7^ cells/mL, falling within this range, was used in studies of glucose, cycloheximide, and Cu^2+^ reported above. Ta et al. studied the effects of initial yeast concentration on the immobilization of *S. cerevisiae* cells on water hyacinth stem pieces [62]. As reported, the immobilized cell density was the highest at an initial concentration of 3 × 10^7^ cfu/mL, and it decreased as the initial concentration was either reduced to 1 × 10^7^ cfu/mL or increased to 7 × 10^7^ cfu/mL [62].

## 4. Conclusions

This study presents a custom-designed microfluidic device that generates micro-droplets for encapsulating yeasts in varying concentrations of glucose, cycloheximide, Cu^2+^, and initial yeast suspension. The growth of *S. cerevisiae* cells within these distinct micro-environments was monitored over an 8 h period. The results indicated that an optimal glucose concentration at around 1% stimulated yeast growth, while the presence of cycloheximide above 0.5 mg/mL and Cu^2+^ higher than 100 μM inhibited it. The growth was also influenced by the initial yeast concentrating, suggesting that both low and high initial concentrations suppressed yeast growth. The optical initial concentration was found to be between 1.67 × 10^7^ and 2.91 × 10^7^ cells/mL. These findings offer valuable insights for improving yeast cultivation in the food industry, biotechnology, biofuel production, and various scientific research applications. Additionally, this platform can be adapted for high-throughput studies of more complex animal and human cells at the single-cell level.

## Data Availability

Data will be made available upon reasonable request.
